# Optimization of Large-Scale Culture Conditions for the Production of Cordycepin with *Cordyceps militaris* by Liquid Static Culture

**DOI:** 10.1155/2014/510627

**Published:** 2014-06-23

**Authors:** Chao Kang, Ting-Chi Wen, Ji-Chuan Kang, Ze-Bing Meng, Guang-Rong Li, Kevin D. Hyde

**Affiliations:** ^1^The Engineering and Research Center of Southwest Bio-Pharmaceutical Resources, Ministry of Education, Guizhou University, Guiyang, Guizhou 550025, China; ^2^Institute of Biology, Guizhou Academy of Sciences, Guiyang, Guizhou 550009, China; ^3^Institute of Excellence in Fungal Research, School of Science, Mae Fah Luang University, Chiang Rai 57100, Thailand; ^4^Botany and Microbiology Department, College of Science, King Saud University, Riyadh 11442, Saudi Arabia

## Abstract

Cordycepin is one of the most important bioactive compounds produced by species of* Cordyceps sensu lato*, but it is hard to produce large amounts of this substance in industrial production. In this work, single factor design, Plackett-Burman design, and central composite design were employed to establish the key factors and identify optimal culture conditions which improved cordycepin production. Using these culture conditions, a maximum production of cordycepin was 2008.48 mg/L for 700 mL working volume in the 1000 mL glass jars and total content of cordycepin reached 1405.94 mg/bottle. This method provides an effective way for increasing the cordycepin production at a large scale. The strategies used in this study could have a wide application in other fermentation processes.

## 1. Introduction

*Cordyceps militaris* is an entomopathogenic fungus belonging to Ascomycota, Sordariomycetidae, Hypocreales, and Cordycipitaceae [1] and is one of the most important traditional Chinese medicinal mushrooms.* Cordyceps militaris* is the type species of* Cordyceps*, which internally parasitizes larva or pupa of lepidopteran insects and forms fruiting bodies on their insect hosts.* Cordyceps militaris* has long been recognized as a desirable alternative for natural* Ophiocordyceps sinensis *[2] as it has been given Chinese Licence number Z20030034/35. This is because the gathering of* Ophiocordyceps sinensis *is causing substantial reductions in populations [3].* Cordyceps militaris* produces many bioactive compounds, including polysaccharides, cordycepin, adenosine, amino acid, organic selenium, ergosterol, sterols, cordycepic acid, superoxide dismutase (SOD), and multivitamins [4, 5].

Cordycepin (3′-deoxyadenosine), a nucleoside analog, was first isolated from* C. militaris* [6] and is one of the species most important biologically active metabolites. It has been regarded as a medicinal agent responsible for immunological regulation [7], anticancer [8], antifungus [9], antivirus [10], antileukemia [11, 12], and antihyperlipidemia [13] activities. Cordycepin is also a Phase I/II clinical stage drug candidate for treatment of refractory acute lymphoblastic leukemia (ALL) patients who express the enzyme terminal deoxynucleotidyl transferase (TdT) (http://www.ClinicalTrials.Gov verified by OncoVista, Inc., 2009).

In previous work, cordycepin has been synthesized by chemical [14, 15] and microbial fermentation using* C. militaris *[6] or* Aspergillus nidulans *[16, 17]. Solid-state fermentation [18, 19], submerged culture [4, 20–24], and surface liquid culture [25–27] have been used in microbial fermentation of cordycepin. Cordycepin obtained through chemistry pathways is hard to purify, and the cost is much higher than through biology fermentation. Thus a major need is to improve the biology methodology [28]. Fermentation time is too long and is difficult to achieve large scale production via solid-state fermentation [18, 19]. Productivity is generally low, the costs are high, and fermentation processes are easily contaminated in submerged culture in large fermenters [4, 20, 21, 29]. Productivity in surface culture techniques is higher as compared to other methods [29, 30] and the cost is lower [23]. New technologies, such as space mutation treatment and high-energy ion beam irradiation, have been used to obtain better Cordycepin producing, novel mutants of* C. militaris*. The resulting mutants were higher cordycepin produces, than the wild strain [30, 31]. Bu et al. [20] reported that the cordycepin in* C. militaris *was substantially increased by the elicitor of* Phytophthora *sp. Research result showed that glucose and yeast extract were effective media components for improved cordycepin production by* C. militaris* [32, 33]. There have been other studies using different culture conditions [21, 24, 25, 32], culture medium, and additives [4, 22–24, 26, 27] for the production of cordycepin via liquid culture. However, as far as we know, these reports studied cordycepin production in 250 mL or 500 mL Erlenmeyer flasks, and there have been no reports to improved cordycepin production using static liquid culture in 1000 mL glass jars. The latter process is a good way to scale up large scale cordycepin production from the laboratory to industry.

In this study, the effects of working volume, carbon sources, nitrogen sources, inorganic salts, growth factor, nucleoside analogue, and amino acid additions were studied in order to improve the cordycepin production by static liquid culture of* C. militaris* (strain CGMCC2459) in 1000 mL glass jars. The results suggested that the optimization medium conditions were helpful for improved large scale cordycepin production.

## 2. Materials and Methods

### 2.1. Microorganism and Seed Culture

The isolate of* C. militaris* (strain CGMCC2459) used in the present study was collected from Mt. Qingcheng in Sichuan Province, China. The microorganism was maintained on potato dextrose agar (PDA) slants. Slants were incubated at 25°C for 7 days and then stored at 4°C. The seed culture was grown in a 250 mL flask containing 70 mL of basal medium (sucrose 20 g/L; peptone 20 g/L; KH_2_PO_4_ 1 g/L; and MgSO_4_ *·*7H_2_O 0.5 g/L) at 25°C on a rotary shaker incubator at 150 rev/min for 5 days [24].

### 2.2. Basal Medium and Static Culture of Glass Jars

The basal medium composition for the fermentation was as follows: sucrose 20 g/L; peptone 20 g/L; KH_2_PO_4_ 1 g/L; and MgSO_4_ *·*7H_2_O 0.5 g/L. The pH was not adjusted, followed by autoclaving for 30 min on the 121°C. The static culture experiments were performed in 1000 mL glass jars (inner diameter 110 mm, height 150 mm) containing basal medium after inoculating with 10% (v/v; the biomass dry weight of seed culture is 54 mg/mL) of the seed culture. The culture was incubated at 25°C without moving for 35 days, and samples were collected at the end of the fermentation from the glass jars for analyzing biomass dry weight and cordycepin production.

### 2.3. Static Culture Conditions

The effects of factors affecting cell growth and the production of cordycepin by* C. militaris* were studied using a one-factor-at-a-time method for static culture. The effects of carbon sources on cordycepinproduction were studied by substituting carbon sources such as sucrose, lactose, soluble starch, and dextrin for glucose at 25°C for 35 days. Effects of nitrogen sources (yeast extract, beef extract, NH_4_NO_3_, NaNO_3_, NH_4_Cl, casein, and carbamide) and inorganic salts (MgCl_2_ *·*6H_2_O, MgSO_4_ *·*7H_2_O, KCl, ZnSO_4_, CaCl_2_ *·*2H_2_O, CaSO_4_ *·*2H_2_O, FeSO_4_ *·*7H_2_O, and K_2_HPO_4_ *·*3H_2_O) were also studied using static culture. Growth factors (Vitamin B_1_ (VB_1_), Vitamin B_6_ (VB_6_), Vitamin B_7_ (VB_7_), Vitamin B_11_ (VB_11_), *α*-naphthylacetic acid (NAA), 3-Indoleacetlc acid (IAA), and 2,4-dichlorophenoxyacetic acid (2,4-D)) were supplemented for 10 mg/L in basal media. Nucleoside analogues (1 g/L) and amino acids (8 g/L) established in our previous study [23] as an initial concentration were separately added to the optimal concentration of carbon and nitrogen source, inorganic salts, and growth factors and cultivated at 25°C for 35 days. All experiments were carried out at triplicate, and mean of results is presented.

### 2.4. Analytical Methods

Samples collected at 35 days from the glass jars were centrifuged at 2810 ×g for 20 min. The mycelium at the bottom of tubes was washed sufficiently with a large amount of distilled water and dried to a constant dry weight at 55°C.

For analysis of extracellular cordycepin, the resulting culture filtrate was obtained by centrifugation at 2810 ×g for 20 min. The supernatant was filtered through a 0.45 *μ*m membrane and the filtrate was analyzed by HPLC (1100 series, Agilent Technology, USA). Accurate quantities of cordycepin (Sigma, USA) were dissolved in distilled water, to give various concentrations for calibration. The mobile phase was 10 mmol/L KH_2_PO_4_, which was dissolved in methanol/distilled water (6 : 94). Elution was performed at a flow rate of 1.0 mL/min with column temperature at 45°C and UV wavelength of 259 nm. Mean values were computed from triplicate samples.

### 2.5. Plackett-Burman Design

The Plackett-Burman design, an effective technique for medium-component optimization [35, 36], was used to select factors that significantly influenced hydrogen production. Sucrose (*X*_1_), peptone (*X*_2_), K_2_HPO_4_ *·*3H_2_O (*X*_4_), MgSO_4_ *·*7H_2_O (*X*_5_), and VB_1_ (*X*_6_) were investigated as key ingredients affecting cordycepin production. Based on the Plackett-Burman design, a 15-run was applied to evaluate eleven factors (including two virtual variables). Each factor was prepared in two levels: −1 for low level and +1 for high level.  Table 1 illustrates the variables and their corresponding levels used in the experimental design. The values of two levels were set according to our preliminary experimental results. The Plackett-Burman design and the response value of cordycepin production are shown in Table 2.

### 2.6. Response Surface Methodology

Response surface methodology using a central composite design was applied to batch cultures of* C. militaris*, for identifying the effects of process variables [35, 36]. In this study, the basic nutrient (carbon sources, nitrogen sources, inorganic salts, and growth factors) and additives (amino acid, nucleoside analogue) were studied for cordycepin production using static liquid culture. In the first test, a three-factor, five-level central composite design with 20 runs was employed. Tested variables (sucrose, K_2_HPO_4_ *·*3H_2_O, and MgSO_4_ *·*7H_2_O) were denoted as *X*_1_, *X*_4_, and *X*_6_, respectively, and each of them was assessed at five different levels, combining factorial points (−1, −1), axial points (−1.6818, +1.6818), and central point (0), as shown in Table 3. Based on the above results, another test, a three-factor, five-level central composite design with 20 runs was employed. Tested variables (amino acid, nucleoside analogue, and culture time) were denoted as *A*, *B*, and *C*, respectively, and each of them was assessed at five different levels, combining factorial points (−1, +1), axial points (−1.6818, +1.6818), and central point (0), as shown in Table 4.

### 2.7. Statistical Analysis

Dry weight and cordycepin production are expressed as means ± SD. An analysis of variance (ANOVA) followed by Tukey's test was applied for multiple comparisons of significant analyses at *P* < 0.05. Statistical data analyses were performed in SPSS version 17.0 software packet. Design-Expert Version 8.0.5b software package (Stat-Ease Inc., Minneapolis, USA) was used for designing experiments as well as for regression and graphical analysis of the experimental data obtained.

## 3. Results and Discussion

### 3.1. Effects of Working Volume on the Biomass and Cordycepin Production

Dissolved oxygen concentration is the key factor in the medium for cell growth and metabolite biosynthesis [21]. Dissolved oxygen does not only have an important function in the respiratory chain, but also in metabolite composition [37, 38]. A previous study showed that the highest cordycepin production and productivity were obtained at lower dissolved oxygen levels [21]. Masuda et al. [25] also reported that a lower medium depth was most efficient for cordycepin production in* C. militaris* by surface culture.

**Figure 1:**
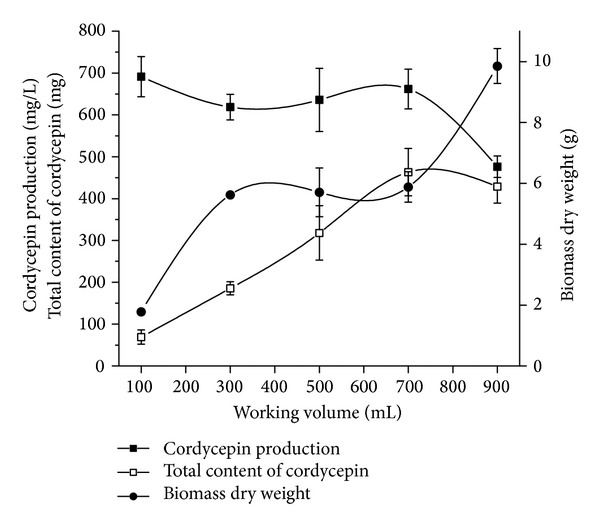
Effects of working volume on the production of cordycepin, total production of cordycepin, and biomass dry weight (total content ofcordycepin (mg) = cordycepin production (mg/L) × working volume (mL)).

In this study, we tried to establish the most efficient working volume of medium for improved cordycepin production. Cultures of* C. militaris* were prepared at the working volumes of 100 to 900 mL (corresponding to a medium depth of 1.26 to 11.31 cm). As shown in Figure 1, cordycepin production reduced gradually with increasing working volume of the medium, from 100 to 700 mL. However, there was no significant difference in cordycepin production in different working volumes. Obviously, the highest working volume of 900 mL did not help in cordycepin production. The result indicates that there is an upper dissolved oxygen limit in the medium for cordycepin production [21]. Lower working volumes result in higher cordycepin productivity, with the highest peak (463.33 ± 56.72 mg of cordycepin) produced at using 700 mL of media. Changes in biomass values were small (between 300 and 700 mL) because of the restricted area and thickness of the mycelial mat. In order to obtain higher cordycepin production, the most effective medium amount was 700 mL (corresponding to an 8.8 cm medium depth) and used as the media volume for next experiment.

### 3.2. Effects of Carbon and Nitrogen Sources on Cordycepin Production

**Figure 2:**
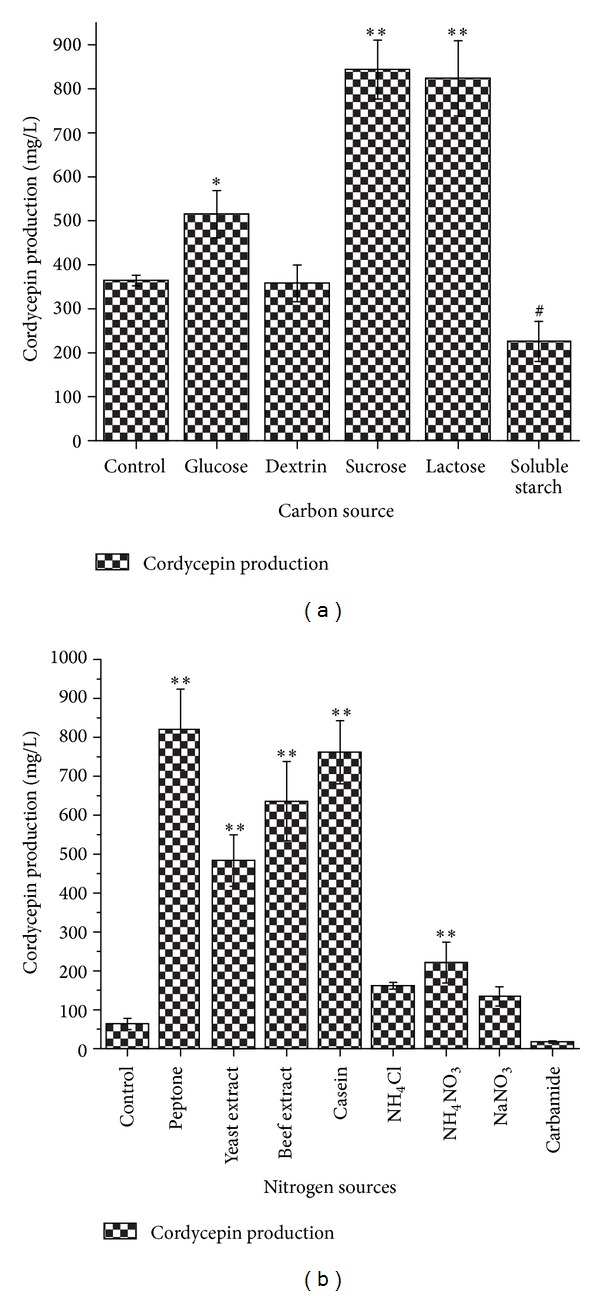
Effects of carbon sources and nitrogen sources on the production of cordycepin: carbon sources (a); nitrogen sources (b); *5% significance level (test group versus control group); **1% significance level (test group versus control group); ^#^5% significance level (control group versus test group).

To find a suitable carbon source for* C. militaris* cordycepin production we added various carbon sources at a concentration of 20 g/L to the sugar-free basal medium. Glucose was previously found to be an excellent precursor of cordycepin production [39]. However, as shown in Figure 2(a), sucrose and lactose proved to be better carbon sources for cordycepin production than glucose in this study. Cordycepin production reached 843.63 ± 66.70 mg/L of sucrose and 823.72 ± 85.64 mg/L of lactose, respectively. Therefore, sucrose was selected as the main carbon source in the remaining experiment.

In previous work, nitrogen showed a regulating role important in cordycepin production and had two effects [40]. One effect was negative since, in excess,* N* promoted a faster mycelial growth and consequently diverted the source of carbon toward energy and biomass production. The other effect was positive because a moderate input contributed to the maintenance of citric acid productive biomass [40]. To investigate the effect of nitrogen sources on cordycepin production in* C. militaris*, various compounds containing nitrogen (inorganic and organic nitrogen) were added individually to nitrogen free basal medium at a concentration of 20 g/L. Among the 8 nitrogen sources tested, peptone, yeast extract, beef extract, casein, and NH_4_NO_3_ were favorable to the cordycepin production (Figure 2(b)). Organic nitrogen was advantageous to both growth and biosynthesis of metabolites. The result is consistent with the experimental data reported [18] and showed that maximum cordycepin production resulted when the peptone was used as a nitrogen source.

### 3.3. Effects of Inorganic Salt and Growth Factor on the Cordycepin Production

**Figure 3:**
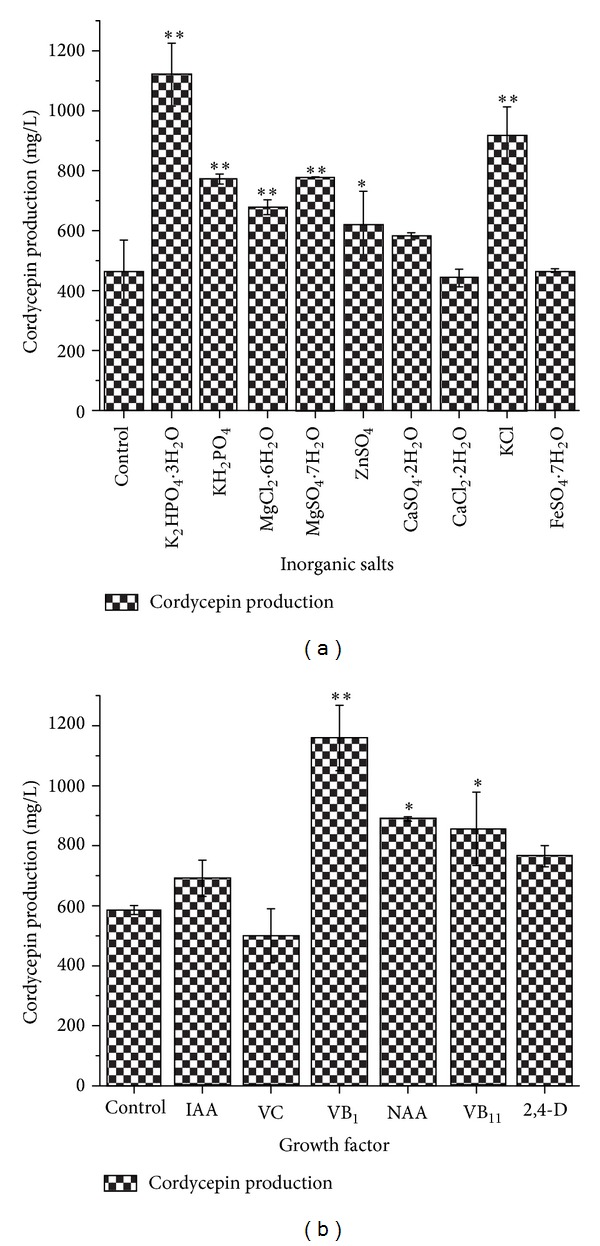
Effects of inorganic salt and growth factors on the production of cordycepin: inorganic salt (a); growth factors (b); *5% significance level (test group versus control group); **1% significance level (test group versus control group).

Inorganic ion was one of the most important nutrition components of medium for the mycelial growth [41]. In order to investigate the effects of inorganic salt for the cordycepin production in* C. militaris*, we tested nine types (at 1 g/L) of inorganic salts (Figure 3(a)). Media with only 20 g/L glucose and 20 g/L peptone were used as the control. The highest cordycepin production (1120.30 ± 105.28 mg/L) by* C. militaris *was observed in medium, when K_2_HPO_4_ *·*3H_2_O was used as an inorganic salt. KH_2_PO_4_, MgSO_4_ *·*7H_2_O, KCl, and MgCl_2_ *·*6H_2_O were also useful inorganic salts. At last, MgSO_4_ *·*7H_2_O and K_2_HPO_4_ *·*3H_2_O were recognized as favorable bioelements for production of cordycepin.

Growth factor is essential for growth response and metabolite production [42]. In order to find the optimal growth factor for cordycepin production,* C. militaris* was cultured in a basal medium with different vitamins and plant growth hormones in static liquid culture. Cordycepin production increased in media with added 10 mg/L of VB_1_, NAA, and VB_11_ (Figure 3(b)). Maximum cordycepin production (1159.34 ± 109.01 mg/L) occurred when VB_1_ was used as the growth factor.

### 3.4. Screening of Important Variables Using Plackett-Burman Design

The data (Table 2) indicated wide variation in cordycepin production in the 15 tests. The data suggested that process optimization is important for improving the efficiency of cordycepin production. Analysis of the regression coefficients and *t* values of 7 factors (Table 5) showed that *X*_1_, *X*_2_, *X*_4_, and *X*_5_ had positive effects on cordycepin production. *X*_6_ had negative effects. The variable affects with a confidence level above 95% are considered as significant factors. Based on these results, three factors (*X*_1_, sucrose; *X*_4_, K_2_HPO_4_ *·*3H_2_O; and *X*_6_, MgSO_4_ *·*7H_2_O) were considered as significant for cordycepin production by static liquid culture methodology.

### 3.5. Optimization by Response Surface Methodology for Carbon Sources and Inorganic Salts

In order to evaluate the influence of medium component on cordycepin production, sucrose, K_2_HPO_4_ *·*3H_2_O, and MgSO_4_ *·*7H_2_O should be examined. The levels of variables for central composite design experiments were selected according to the above results of Plackett-Burman design.  Table 6 shows the detailed experimental design and results. Regression analysis was performed to fit the response function (cordycepin production) with the experimental data. From the variables obtained (Table 6), the model is expressed by (1), which represents cordycepin production (*Y*_1_) as a function of sucrose (*X*_1_), K_2_HPO_4_ *·*3H_2_O (*X*_4_), and MgSO_4_ *·*7H_2_O (*X*_6_) concentrations:

(1) Y1=1419.68−98.95X1+31.45X4−51.68X6 −16.89X1X4−20.48X1X6+2.36X4X6 −106.03X12−55.06X42−150.31X62.

Results of *F*-test analysis of variance (ANOVA) showed that the regression was statistically significant at 95% and 99% confidence levels (Table 7). The “*F* value” of the model was 9.21, and the value of “Prob > *F*” < 0.01 indicated that the model was significant. In this case, linear terms of *X*_1_ and quadratic terms of *X*_1_^2^, *X*_4_^2^, *X*_6_^2^ were significant of model terms for cordycepin production. The “*Lack of Fit F value*” of 0.0903 implied that the “*Lack of Fit*” was not significant relative to the pure error (*P* > 0.05). The Pred-*R* ^2^ of 0.3183 was not as close to the Adj-*R* ^2^ of 0.7954 as one might normally expect. The result suggested that some factors were not considered in the model. However, the “Adeq Precision” of 8.173 indicated that the model was adequate for prediction production of cordycepin.

**Figure 4:**
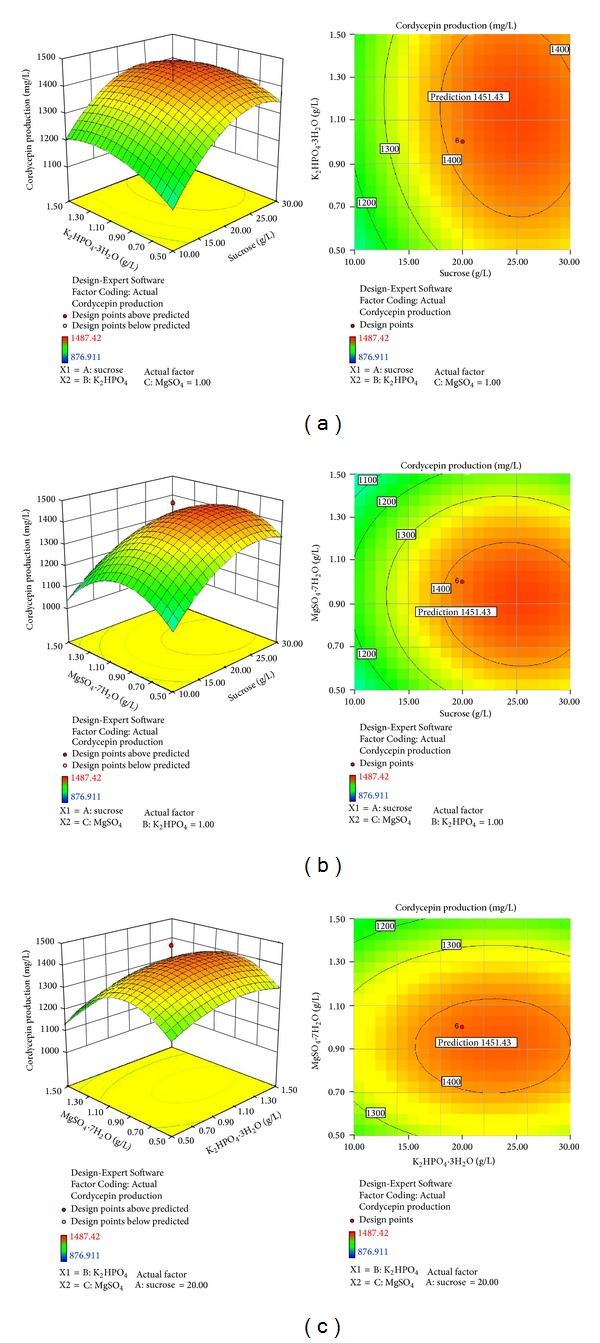
Three-dimensional response surface plots and two-dimensional contour plots for cordycepin production by* C. militaris* (strain CGMCC2459) showing variable interactions of (a) sucrose and K_2_HPO_4_ *·*3H_2_O; (b) sucrose and MgSO_4_ *·*7H_2_O; (c) K_2_HPO_4_ *·*3H_2_O and MgSO_4_ *·*7H_2_O.

The response surface plot obtained from (1) is shown in Figure 4. It is evident that cordycepin production reached its maximum at a combination of coded level (*X*_1_, sucrose, level 0.47; *X*_4_, K_2_HPO_4_ *·*3H_2_O, level 0.21; *X*_6_, MgSO_4_ *·*7H_2_O, level −0.20) when using canonical analysis of the Design-Expert Version 8.0.5b software package. The model predicted a maximum response of 1451.43 mg/L cordycepin production at levels of sucrose 24.7 g/L, K_2_HPO_4_ *·*3H_2_O 1.11 g/L, and MgSO_4_ *·*7H_2_O 0.90 g/L as optimized medium components.

### 3.6. Effects of Nucleoside Analogue and Amino Acid on the Production of Cordycepin

**Figure 5:**
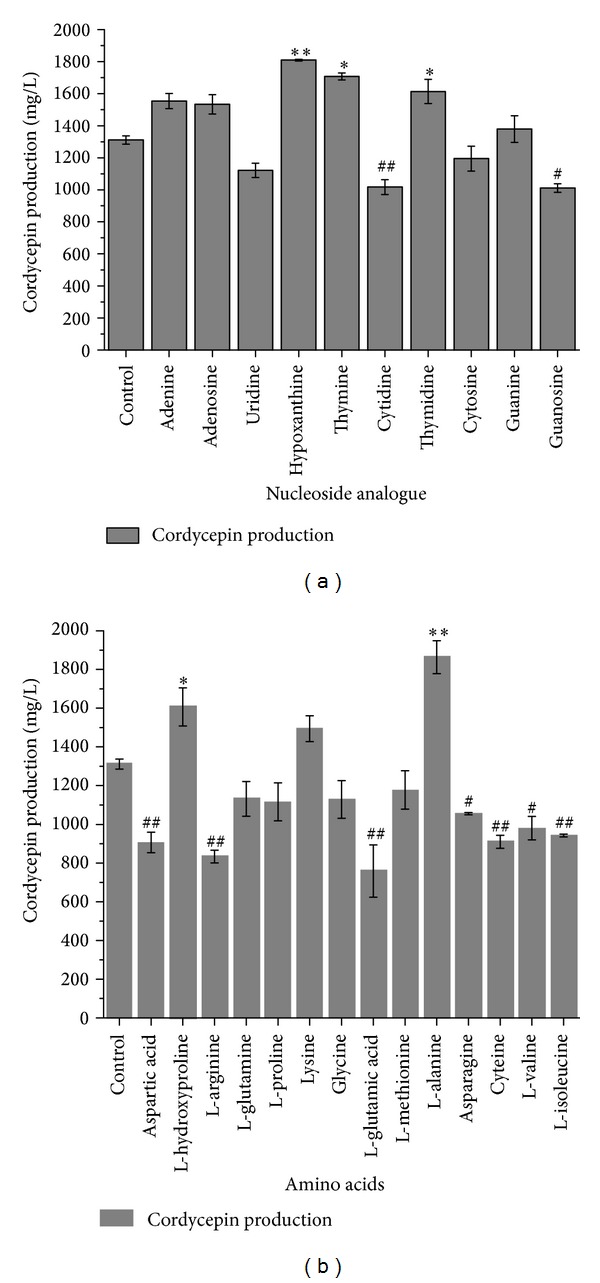
Effects of nucleoside analogue and amino acid on the production of cordycepin: nucleoside analogue (a); amino acid (b); *5% significance level (test group versus control group); **1% significance level (test group versus control group); ^#^5% significance level (control group versus test group); ^##^1% significance level (control group versus test group).

Chassy and Suhadolnik [43] reported that adenine and adenosine were precursors for cordycepin synthesis. Amino acids were regarded as the best substance for improved cordycepin production [4, 23]. Based on these results, among 10 different kinds of nucleoside analogue were supplemented for 1 g/L in this study. As shown in Figure 5(a), cordycepin production increased obviously in the medium with hypoxanthine, thymine, and thymidine additives. The highest production of cordycepin was achieved, when hypoxanthine was used as the nucleoside analogue. Hypoxanthine's molecular structure is similar to purine bases found in cordycepin. Substituent on purine bases structure is –OH on hypoxanthine rather than –NH_2_. The –OH should be replaced in metabolic pathways. In addition, among 14 different amino acids were tested for 8 g/L. As shown in Figure 5(b), L-alanine can improve cordycepin production. Previous research showed that adenine, adenosine, and glycine were good additives for increased cordycepin production [4, 23, 26, 27]. L-alanine may be an important nutritional element for* C. militaris* or component of cordycepin production. Hypoxanthine and L-alanine were the best additives to promote cordycepin production in this study.

### 3.7. Optimization by Response Surface Methodology for Amino Acid, Nucleoside Analogue, and Fermentation Time

**Figure 6:**
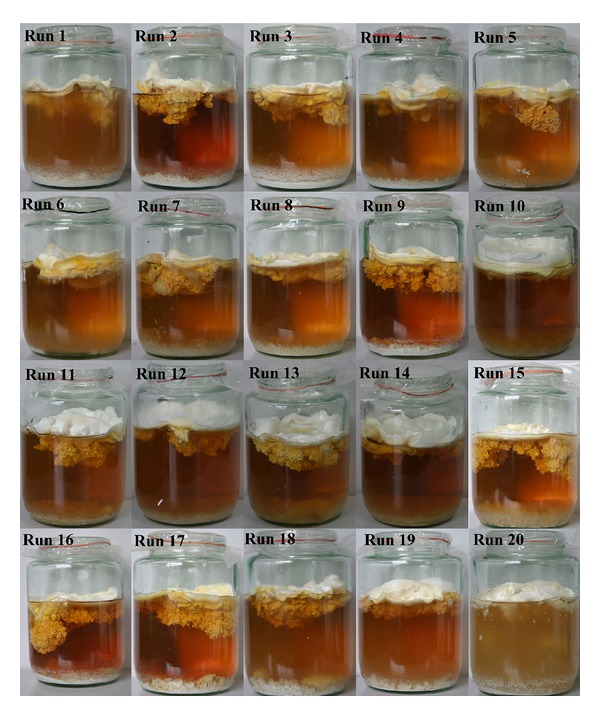
Morphology of* C. militaris* (strain CGMCC2459) in 700/1000 mL glass jars at the end of the fermentation process by response surface methodology: symbols in photos indicated 20 runs.

Similarly, central composite design was also applied to study the significant factors (hypoxanthine, L-alanine, and culture time) and their optimal levels.  Figure 6 shows the morphological characteristics of* C. militaris* in 1000 mL glass jars after fermentation by static liquid fermentation.  Table 8 shows the detailed experimental design and results. Regression analysis was performed to fit the response function (cordycepin production) with the experimental data. From the variables obtained (Table 9), the model was expressed by (2), which represented cordycepin production (*Y*_2_) as a function of hypoxanthine (*A*), L-alanine (*B*), and culture time (*C*, time), concentrations:

(2) Y2=1991.13−10.05A+10.70B−151.02C +2.81AB−183.77AC−26.14BC −146.09A2−146.03B2−371.65C2.  

Results of *F*-test analysis of variance (ANOVA) showed that the regression was statistically significant at 95% and 99% confidence level (Table 9). The “*F* value” of the model was 11.91, and the value of “Prob > *F*” < 0.01 indicated that the model was significant. In this case, linear terms of *C*; interactive terms of *AC*; and quadratic terms of  *A* ^2^, *B* ^2^, and *C* ^2^ were significant in model terms for cordycepin production. The “*Lack of Fit F value*” of 0.1707 implied that the “*Lack of Fit*” was not significant relative to the pure error (*P* > 0.05). The Pred-*R* ^2^ of 0.4162 was not as close to the Adj-*R* ^2^ of 0.8378 as one might normally expect. The result suggested that some factors were not also considered in the model. However, the “Adeq Precision” of 11.222 indicated that the model was adequate for prediction production of cordycepin.

**Figure 7:**
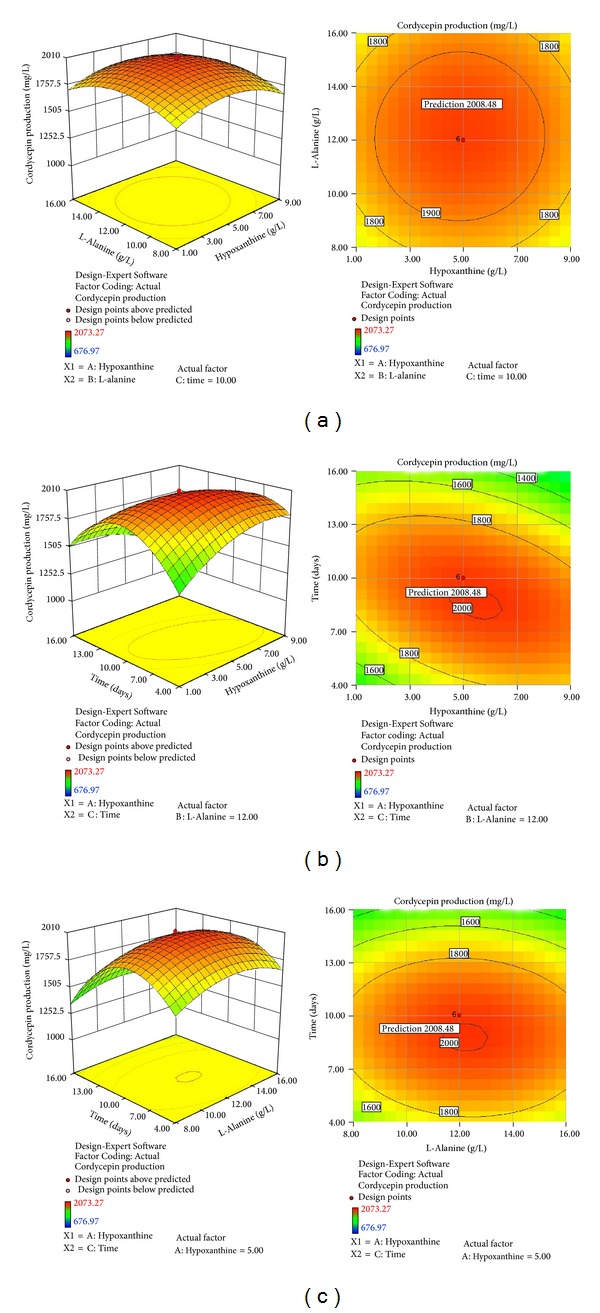
Three-dimensional response surface plots and two-dimensional contour plots for cordycepin production by* C. militaris* (strain CGMCC2459) showing variable interactions of (a) hypoxanthine and L-alanine; (b) hypoxanthine and time; (c) L-alanine and time.

The response surface plot obtained from (2) is shown in Figure 7. It is evident that cordycepin production reached its maximum with a combination of coded level (*A*, hypoxanthine, level 0.11; *B*, L-alanine, level 0.06; *C*, time, level −0.23) by canonical analysis of the Design-Expert Version 8.0.5b software package. The model predicted a maximum response of 2008.48 mg/L cordycepin production at levels of hypoxanthine 5.45 g/L, L-alanine 12.23 g/L, and time 8.6 days (in the practical test 8 days) as optimized medium components.

In previous work, the orthogonal design method [44–46], Box-Behnken design [34, 47], and central composite design [31] were used to optimize culture conditions for cordycepin production by* Cordyceps *sp. These experimental designs have been successfully used to optimize medium for the mycelial growth and microbial metabolite production in liquid culture processes. In this study, static liquid culture conditions are optimized for the cordycepin production using response surface methodology and are an effective way to enhance the productivity of cordycepin and biomass in* C. militaris*.

### 3.8. Verification Experiments and Batch Culture

**Figure 8:**
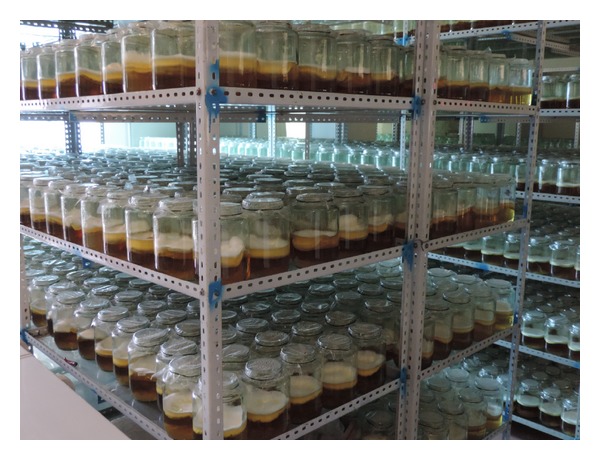
Batch culture for cordycepin production under optimized culture conditions by static liquid culture using* C. militaris* (strain CGMCC2459).

Based on the results of response surface methodology, the optimized medium was prepared as follows: peptone 20 g/L; sucrose 24.7 g/L; K_2_HPO_4_ *·*3H_2_O 1.11 g/L; MgSO_4_ *·*7H_2_O 0.90 g/L; VB_1_ 10 mg/L; hypoxanthine 5.45 g/L; and L-alanine 12.23 g/L. Five experiments were performed to confirm the above optimal culture requirements. The data were 2011.15 mg/L, 2000.69 mg/L, 1989.22 mg/L, 1969.6 mg/L, and 2061.37 mg/L, respectively. The average cordycepin production was 2006.41 ± 34.37 mg/L. The experimental values were particularly close to the predicted values (2008.48 mg/L). The result confirmed the model suited the predictive of hyperproduction of cordycepin by* C. militaris* in static liquid culture. Batch culture was carried for cordycepin production under optimized culture conditions (Figure 8).

### 3.9. *In Vitro* Cordycepin Production Using Liquid Culture in Other Studies

The highest report for cordycepin production was 14300 mg/L by Masuda et al. [29] (Table 10). In our experiment, cordycepin production at 2008.48 mg/L was lower. However, a maximum total content of cordycepin (1405.94 mg) was achieved in our study. This is a second higher report of cordycepin production in one single fermenter. The results showed that the culture conditions will provide an effective way for increasing cordycepin production.

## 4. Conclusion

In this work, single factor design, Plackett-Burman design, and central composite design were employed to establish the key factors and identify optimal culture conditions which improved cordycepin production by* C. militaris* CGMCC2459. Optimal media contained peptone 20 g/L; sucrose 24.7 g/L; K_2_HPO_4_ *·*3H_2_O 1.11 g/L; MgSO_4_ *·*7H_2_O 0.90 g/L; VB_1_ 10 mg/L; hypoxanthine 5.45 g/L; and L-alanine 12.23 g/L. Hypoxanthine and L-alanine were added to the optimal medium at 8.6 days. Optimal incubation conditions were 25°C at an unaltered pH of 35 days. Using these culture conditions, a maximum production of cordycepin was 2008.48 mg/L for 700 mL working volume in the 1000 mL glass jars, and total content of cordycepin reached 1405.94 mg/bottle (700 mL/1000 mL). This method provides an effective way for increasing the cordycepin production at a large scale. The strategies used in this study could have a wide application in other fermentation process.

## Figures and Tables

**Table 1:** Range of different factors investigated with Plackett-Burman design.

Symbol	Variables	Experimental value	
		Low (−1)	High (+1)
*X*_1_	Sucrose (g/L)	20	25
*X*_2_	Peptone (g/L)	20	25
*X*_3_	Virtual 1	−1	1
*X*_4_	K_2_HPO_4_ *·*3H_2_O (g/L)	1	1.25
*X*_5_	VB_1_ (g/L)	10	12.5
*X*_6_	MgSO_4_ *·*7H_2_O (g/L)	1	1.25
*X*_7_	Virtual 2	−1	1

**Table 2:** Plackett-Burman design and response values.

Runs	Experimental value							*Y* (mg/L) Cordycepin production
	*X*_1_	*X*_2_	*X*_3_	*X*_4_	*X*_5_	*X*_6_	*X*_7_	
1	1	−1	1	−1	−1	−1	1	812.36 ± 26.83
2	1	1	−1	1	−1	−1	−1	1395.18 ± 8.4
3	−1	1	1	−1	1	−1	−1	900.25 ± 10.29
4	1	−1	1	1	−1	1	−1	802.45 ± 45.43
5	1	1	−1	1	1	−1	1	1097.66 ± 25.57
6	1	1	1	−1	1	1	−1	845.87 ± 24.94
7	−1	1	1	1	−1	1	1	786.35 ± 7.61
8	−1	−1	1	1	1	−1	1	805.08 ± 29.1
9	−1	−1	−1	1	1	1	−1	920.48 ± 16.21
10	1	−1	−1	−1	1	1	1	694.01 ± 79.51
11	−1	1	−1	−1	−1	1	1	497.28 ± 4.44
12	−1	−1	−1	−1	−1	−1	−1	592.83 ± 16.13
13	0	0	0	0	0	0	0	1134.14 ± 2.59
14	0	0	0	0	0	0	0	1100.21 ± 0.08
15	0	0	0	0	0	0	0	1133.56 ± 1.85

**Table 3:** Factors and levels of central composite design for carbon sources and inorganic salts.

Symbol	Variables	Code level				
		−1.6818	−1	0	1	1.6818
*X*_1_	Sucrose (g/L)	3.1821	10	20	30	36.8179
*X*_4_	K_2_HPO_4_ *·*3H_2_O (g/L)	0.1591	0.5	1	1.5	1.8409
*X*_6_	MgSO_4_ *·*7H_2_O (g/L)	0.1591	0.5	1	1.5	1.8409

**Table 4:** Factors and levels of central composite design for amino acid, nucleoside analogue, and time.

Symbol	Variables	Code level				
		−1.6818	−1	0	1	1.6818
*A*	Hypoxanthine (g/L)	0.53	1	5	9	10.53
*B*	L-alanine (g/L)	5.27	8	12	16	18.72
*C*	Culture time (days)	−0.09	4	10	16	20.09

**Table 5:** Results of regression analysis of Plackett-Burman design.

Symbol	Regression analysis				
	Effect	Coefficient	Standard error	*T*	*P*
		845.82	32.86	25.74	0.000**
*X*_1_	190.88	95.44	32.86	2.90	0.027*
*X*_2_	149.23	74.62	32.86	2.27	0.064
*X*_3_	−40.85	−20.42	32.86	−0.62	0.557
*X*_4_	244.10	122.05	32.86	3.71	0.010*
*X*_5_	62.81	31.41	32.86	0.96	0.376
*X*_6_	−176.15	−88.08	32.86	−2.68	0.037*
*X*_7_	−127.39	−63.69	32.86	−1.94	0.101
Ct Pt		276.82	73.48	3.77	0.009**

*5% significance level; **1% significance level; *X*_1_–*X*_7_ are symbols shown in Table 1.

**Table 6:** Experimental design and responses of the central composite design for carbon sources and inorganic salts.

Run	Variables Code			*Y* (mg/L) Cordycepin production	Run	Variables Code			*Y* (mg/L) Cordycepin production
	*X*_1_	*X*_4_	*X*_6_			*X*_1_	*X*_4_	*X*_6_	
1	−1	−1	−1	1080.55 ± 109.69	11	0	0	0	1399.43 ± 124.44
2	1	−1	−1	1359.48 ± 12.61	12	0	0	0	1415.43 ± 42.13
3	−1	1	−1	1158.59 ± 12.15	13	0	0	0	1487.42 ± 16.38
4	1	1	−1	1289.90 ± 47.00	14	0	0	0	1409.43 ± 155.22
5	−1	−1	1	980.55 ± 34.72	15	−1.6818	0	0	876.91 ± 16.69
6	1	−1	1	1097.48 ± 52.42	16	1.6818	0	0	1290.00 ± 14.71
7	−1	1	1	987.95 ± 2.89	17	0	−1.6818	0	1110.57 ± 157.63
8	1	1	1	1117.38 ± 116.84	18	0	1.6818	0	1344.66 ± 65.54
9	0	0	0	1485.38 ± 12.19	19	0	0	−1.6818	958.39 ± 224.14
10	0	0	0	1333.48 ± 94.22	20	0	0	1.6818	958.00 ± 75.82

**Table 7:** ANOVA for response surface quadratic polynomial model for carbon sources and inorganic salts.

Source	Sum of quares	df	Mean Square	*F*-value	*P*-value Prob > *F*
*Model *	6.507*E* + 005	9	72300.77	9.21	0.0009**
*X*_1_-*X*_1_	1.337*E* + 005	1	1.337*E* + 005	17.03	0.0021**
*X*_4_-*X*_4_	13504.30	1	13504.30	1.72	0.2190
*X*_6_-*X*_6_	36477.48	1	36477.48	4.65	0.0565
*X*_1_ *X*_4_	2282.55	1	2282.55	0.29	0.6016
*X*_1_ *X*_6_	3356.89	1	3356.89	0.43	0.5279
*X*_4_ *X*_6_	44.38	1	44.38	5.653*E* − 003	0.9416
*X*_1_^2^	1.620*E* + 005	1	1.620*E* + 005	20.63	0.0011**
*X*_4_^2^	43685.18	1	43685.18	5.56	0.0400**
*X*_6_^2^	3.256*E* + 005	1	3.256*E* + 005	41.47	*<*0.0001∗∗
Residual	78513.73	10	7851.37		
*Lack of Fit *	61670.32	5	12334.06	3.66	0.0903
*Pure Error *	16843.41	5	3368.68		
Cor Total	7.292*E* + 005	19			

*R* ^2^ = 0.8923; CV = 7.34%; Pred-*R* ^2^ = 0.3183; Adj-*R* ^2^ = 0.7954; Adeq Precision = 8.173; ∗5% significance level; ∗∗1% significance level.

**Table 8:** Experimental design and responses of the central composite design for amino acid, nucleoside analogue, and time.

Run	Variables Code			*Y* (mg/L) Cordycepin production	Run	Variables Code			*Y* (mg/L) Cordycepin production
	*A*	*B*	*C*			*A*	*B*	*C*	
1	−1	−1	−1	1383.01 ± 41.53	11	0	0	0	2041.25 ± 54.70
2	1	−1	−1	1422.52 ± 39.41	12	0	0	0	2020.97 ± 73.70
3	−1	1	−1	1216.88 ± 8.69	13	0	0	0	1998.18 ± 49.48
4	1	1	−1	1857.51 ± 164.86	14	0	0	0	2068.60 ± 72.79
5	−1	−1	1	1216.87 ± 253.38	15	−1.6818	0	0	1590.14 ± 222.14
6	1	−1	1	1111.18 ± 170.50	16	1.6818	0	0	1573.90 ± 776.16
7	−1	1	1	1536.05 ± 75.17	17	0	*−*1.6818	0	1636.44 ± 65.23
8	1	1	1	851.70 ± 17.01	18	0	1.6818	0	1527.98 ± 177.46
9	0	0	0	2073.27 ± 65.85	19	0	0	−1.6818	1211.14 ± 82.58
10	0	0	0	1743.09 ± 14.81	20	0	0	1.6818	676.97 ± 142.74

**Table 9:** ANOVA for response surface quadratic polynomial model for amino acid, nucleoside analogue, and time.

Source	Sum of quares	df	Mean Square	*F-*value	*P-*value Prob > *F*
*Model *	2.899*E* + 006	9	3.221*E* + 005	11.91	0.0003∗∗
*A*-*A*	1378.39	1	1378.39	0.051	0.8260
*B*-*B*	1564.06	1	1564.06	0.058	0.8148
*C*-*C*	3.115*E* + 005	1	3.115*E* + 005	11.51	0.0068∗
*AB*	63.06	1	63.06	2.331*E* − 003	0.9624
*AC*	2.702*E* + 005	1	2.702*E* + 005	9.99	0.0102∗
*BC*	5468.49	1	5468.49	0.20	0.6626
*A* ^2^	3.076*E* + 005	1	3.076*E* + 005	11.37	0.0071∗∗
*B* ^2^	3.073*E* + 005	1	3.073*E* + 005	11.36	0.0071∗∗
*C* ^2^	1.990*E* + 006	1	1.990*E* + 006	73.58	<0.0001∗∗
Residual	2.705*E* + 005	10	27053.63		
*Lack of Fit *	1.928*E* + 005	5	38561.98	2.48	0.1707
*Pure Error *	77726.41	5	15545.28		
Cor Total	3.169*E* + 006	19			

*R* ^2^ = 0.9146; CV = 10.70%; Pred-*R* ^2^ = 0.4162; Adj-*R* ^2^ = 0.8378; Adeq Precision = 11.222; ∗5% significance level; ∗∗1% significance level.

**Table 10:** Cordycepin production in the medium by liquid culture in different studies.

No.	Methodology	Working volume of the medium v/v (mL/mL)	Cordycepin production (mg/L)	Total content of cordycepin in one bottle (mg)	References
1	Submerged culture	50/250	245.7	12.5	Mao et al., [32]
2	Submerged culture	50/250	420.5	21.03	Mao and Zhong [21]
3	Surface liquid culture	100/500	640	64	Masuda et al., [25]
4	Shaking + Static	100/250	2214.5	221.45	Shih et al., [34]
5	Surface liquid culture	100/500	2500	250	Masuda et al., [26]
6	Surface liquid culture	100/500	3100	310	Das et al., [30]
7	Surface liquid culture	100/500	8570	857	Das et al., [27]
8	Submerged culture	100/500	1644.21	164.42	Wen et al., [23]
9	Dark + Shaking	100/500	1015	101.5	Kang et al., [24]
10	Surface liquid culture	150/500	14300	2145	Masuda et al., [29]
**11**	**Static liquid culture**	**700/1000**	**2008.48**	**1405.94**	**In this study**
